# Channel Attention GAN-Based Synthetic Weed Generation for Precise Weed Identification

**DOI:** 10.34133/plantphenomics.0122

**Published:** 2024-03-28

**Authors:** Tang Li, Motoaki Asai, Yoichiro Kato, Yuya Fukano, Wei Guo

**Affiliations:** ^1^ Graduate School of Agricultural and Life Sciences, The University of Tokyo, Tokyo 188-0002, Japan.; ^2^ Institute for Plant Protection, National Agriculture and Food Research Organization, Fukushima 960-2156, Japan.; ^3^ Graduate School of Horticulture, Chiba University, Chiba 271-0092, Japan.

## Abstract

Weed is a major biological factor causing declines in crop yield. However, widespread herbicide application and indiscriminate weeding with soil disturbance are of great concern because of their environmental impacts. Site-specific weed management (SSWM) refers to a weed management strategy for digital agriculture that results in low energy loss. Deep learning is crucial for developing SSWM, as it distinguishes crops from weeds and identifies weed species. However, this technique requires substantial annotated data, which necessitates expertise in weed science and agronomy. In this study, we present a channel attention mechanism-driven generative adversarial network (CA-GAN) that can generate realistic synthetic weed data. The performance of the model was evaluated using two datasets: the public segmented Plant Seedling Dataset (sPSD), featuring nine common broadleaf weeds from arable land, and the Institute for Sustainable Agro-ecosystem Services (ISAS) dataset, which includes five common summer weeds in Japan. Consequently, the synthetic dataset generated by the proposed CA-GAN obtained an 82.63% recognition accuracy on the sPSD and 93.46% on the ISAS dataset. The Fréchet inception distance (FID) score test measures the similarity between a synthetic and real dataset, and it has been shown to correlate well with human judgments of the quality of synthetic samples. The synthetic dataset achieved a low FID score (20.95 on the sPSD and 24.31 on the ISAS dataset). Overall, the experimental results demonstrated that the proposed method outperformed previous state-of-the-art GAN models in terms of image quality, diversity, and discriminability, making it a promising approach for synthetic agricultural data generation.

## Introduction

Enhancing crop productivity to meet the increasing food demand is a major global challenge today [1]. Weeds are among the most significant biotic constraints on crop production [2]. However, the widespread use of herbicides has led to the evolution of herbicide-resistant species and a loss of weed diversity [3].

Site-specific weed management (SSWM) is a solution for cost-efficient weed management in precision agriculture. It involves weed control only where needed in the field [4]. Implementing deep learning (DL) for weed seedling identification can provide an accurate weed localization system for SSWM. However, the quality of DL largely depends on the size of the datasets used to cope with real-world conditions, and the availability of data is significantly constrained in domain-specific applications, such as plant detection and recognition [5]. Annotation of weed data can be both time-consuming and error-prone, because it relies on specialized knowledge and skills in weed science and agronomy. Therefore, there are limited databases containing images of weeds, which is a major impediment to the development of SSWM.

Data generation, instead of the traditional approach of data acquisition and labeling, can be used to generate realistic image data, i.e., simulate the statistical properties of natural scenarios according to a certain logic and generate a large quantity of artificial data without additional manual annotation [6]. This can largely alleviate the problem of a lack of training data. However, a large quantity and diversity of individual weed data are required to simulate realistic natural scenarios. Traditional data augmentation schemes (e.g., rotation, scale, and added noise) can alleviate the scarcity of individual data to some extent but cannot fundamentally increase the diversity of individual data.

Creating generative models is an alternative method for obtaining individual weed data by generating large and diverse datasets of individual weeds based on relatively limited training data. Generative models have been previously employed to enhance tasks such as plant classification, leaf counting, and leaf segmentation, yielding promising results. Recently, generative models have advanced dramatically with generative adversarial networks [7], leading the way in generating diverse high-fidelity images with models learned directly from data. Prior studies have demonstrated that utilizing GAN-generated synthetic samples for data augmentation can improve the performance of various visual recognition tasks [8].

In contrast, conditional generation achieves control over the content of the output image by specifying the category of generation along with the input noise. Conditional GANs [9] realize this by passing the category information of the input image to the model in a supervised form. In the auxiliary classifier GAN [10], the category information of the image is concatenated in the input noise by means of one-hot encoding, and the discriminator is constrained to determine whether the semantic information contained in the synthetic data is correct by adding an auxiliary classifier. In cGANs with a projection discriminator [11], the discriminator is conditioned using the cosine similarity between its features and a set of learned class embeddings as additional evidence to distinguish between real and generated synthetic samples, effectively encouraging the generation of samples whose features match a learned class prototype.

In the agricultural domain, a growing number of researchers have started to employ GANs. Giuffrida et al. [12] proposed a conditional GAN model called ARIGAN to generate images of *Arabidopsis* plants using formulated leaf counts. They showed that synthetic images for training data augmentation can reduce the absolute difference in leaf counting by 5.4%. Zhu et al. [13] utilized a conditional GAN with a leaf mask to generate plant images. They reported a 16.7% decrease in the absolute counting difference when training data were augmented with synthetic images. Madsen et al. [14] proposed WacGAN by combining WGAN-GP [15] and ACGAN [10] with the plant species as the conditional term. WacGAN can generate nine classes of plant images while ensuring image fidelity and achieved a performance of 58.9% for the multiclass discriminability test. Madsen et al. [16] extended their model by proposing WacGAN-info, which supplemented the GAN configuration with an unsupervised learning branch to control the visual appearance of synthetic data through an additional set of potential input variables. They reported that the generated synthetic samples showed a resemblance to the intended species, achieving an average recognition accuracy of 64.3%. Espejo-Garcia et al. [17] compared the generative effects of DCGAN for generating synthetic tomato and black nightshade images with different network configurations and hyperparameters and evaluated the performance of weed identification with the generated synthetic data used for data augmentation. They reported the best Fréchet inception distance (FID) score of 86.93 for tomato synthesis and 153.44 for black nightshade and obtained promising performances between 90% and 100% for identification accuracy.

Although prior research has demonstrated the potential of GANs for generating synthetic agricultural image data to augment training dataset, the quality and fidelity of the generated synthetic data remain challenging. Despite previous research showing this potential, some previous methods still had pixel errors, and the generated synthetic samples often only vaguely resembled the intended species, resulting in an average recognition accuracy of approximately 64.3%. This level of accuracy is low and can hinder downstream tasks.

We propose a method that addresses these challenges by incorporating a novel architecture that improves the visual quality and discriminability of the generated images. The main contributions of this study are summarized as follows.

1. We propose a channel attention mechanism-based GAN (CA-GAN) architecture designed to close the gap in fidelity, diversity, and classification accuracy between the data generated by GANs and real-world images.
2. We compared our model with other GAN architectures using both the benchmark segmented Plant Seedling Dataset (sPSD) and auxiliary Institute for Sustainable Agro-ecosystem Services (ISAS) dataset.

## Materials and Methods

Our study leverages two datasets: the sPSD, which serves as a benchmark for model comparison, and the ISAS, which is targeted at prevalent weed species in Japan. The sPSD allows us to measure our model’s performance against existing approaches by providing a diverse range of species for a comparative analysis. Meanwhile, the ISAS dataset is curated to focus on the most common weeds in Japan, addressing the specific challenge of early-stage weed detection in this region.

The emphasis on early growth stages is strategic, as early intervention is critical in weed management. Identifying and addressing weed growth at this stage is key to preventing the establishment and spread of invasive species, which can be more difficult to manage later and can cause significant crop yield losses. By targeting the initial growth phases, our research aims to enhance the efficacy of weed control practices.

### Benchmark dataset sPSD

The sPSD [18] served as the primary benchmark dataset, comprising segmented RGB images of plant seedlings from 12 different species cultivated in greenhouse conditions.

To align the dataset with our study’s focus on the early growth stages of weeds, images exceeding a spatial resolution of 400 pixels in any dimension were excluded. This step effectively removed representations of later growth stages, ensuring a dataset composed of early-stage seedlings.

Grass species, except ZEAMX, were also excluded due to segmentation issues as described in [16], resulting in nine plant species being represented in the final dataset (Table 1).

**Table 1.** An overview of plant species and the sample count within the sPSD is provided. Images of grass species (except ZEAMX) and those with a resolution exceeding 400 pixels in either dimension were excluded.

Weed code	Latin name	English name	No. of samples
SNIAR	*Sinapis arvensis*	Charlock	297
GALAP	*Galium aparine*	Cleavers	270
STEME	*Stellaria media*	Common Chickweed	591
CHEAL	*Chenopodium album*	Fat Hen	447
ZEAMX	*Zea mays*	Maize	149
MATIN	*Matricaria perforata*	Scentless Mayweed	498
CAPBP	*Capsella bursa-pastoris*	Shepherd’s Purse	225
GERPU	*Geranium pusillum*	Small-flowered Cranesbill	429
BEAVA	*Beta vulgaris var. altissima*	Sugar Beets	191

### Auxiliary dataset ISAS

The ISAS dataset functioned as an auxiliary dataset to assess the robustness of the model. The data collection was performed in the greenhouse located at the Institute for Sustainable Agro-ecosystem Services (ISAS, 35°44′09″ N and 139°32′24″ E) at the University of Tokyo during the 2018 and 2019 seasons. Five common summer annual weed species in Japan were cultivated independently, with each cavity tray planted with the same species of weed. All seeds were purchased from the ESPEC MIC Corporation (https://www.especmic.co.jp/).

To facilitate data labeling, we cultivated different species of weeds independently, i.e., each cavity tray was planted with the same species of weed. Color checker was used for uniform color correction of images. Furthermore, shade may cause leaf deformation. To ensure that our data were consistent with real-world field data, we utilized 100% natural light conditions during the natural growing season to mimic the growth environment of weeds under field crop conditions.

To generate data targeting the early growth stages of the weeds, data were collected from the cotyledon stage to the fourth true leaf stage. Raw RGB image data were manually photographed and labeled at the detection level using the Labelbox data annotation platform (https://labelbox.com/).

### Preprocessing protocol

The preprocessing protocol was designed to be uniform for both sPSD and ISAS datasets to maintain the aspect ratio of individual weeds and ensure that plants occupied a high proportion of pixels in the image. This protocol involved several steps.

1. Removal of oversized images with dimensions greater than 400 pixels.
2. Padding of images to a standardized size (470 × 470 pixels for both sPSD and ISAS).
3. Cropping of the padded images to a centered frame of 400 × 400 pixels.
4. Resizing the cropped images to a uniform resolution of 128 × 128 pixels, suitable for our CA-GAN model input.

For the ISAS dataset specifically, segmentation was conducted before preprocessing using Easy Plant Canopy Coverage (EasyPCC) version 2 [19]. EasyPCC is a tool designed for high-throughput measurement of the plant canopy coverage ratio, providing a reliable and automated method for analyzing a large number of images under diverse field conditions. This method ensured that our segmentation was both precise and consistent across different lighting conditions and growth stages of the weeds.

**Fig. 1.**
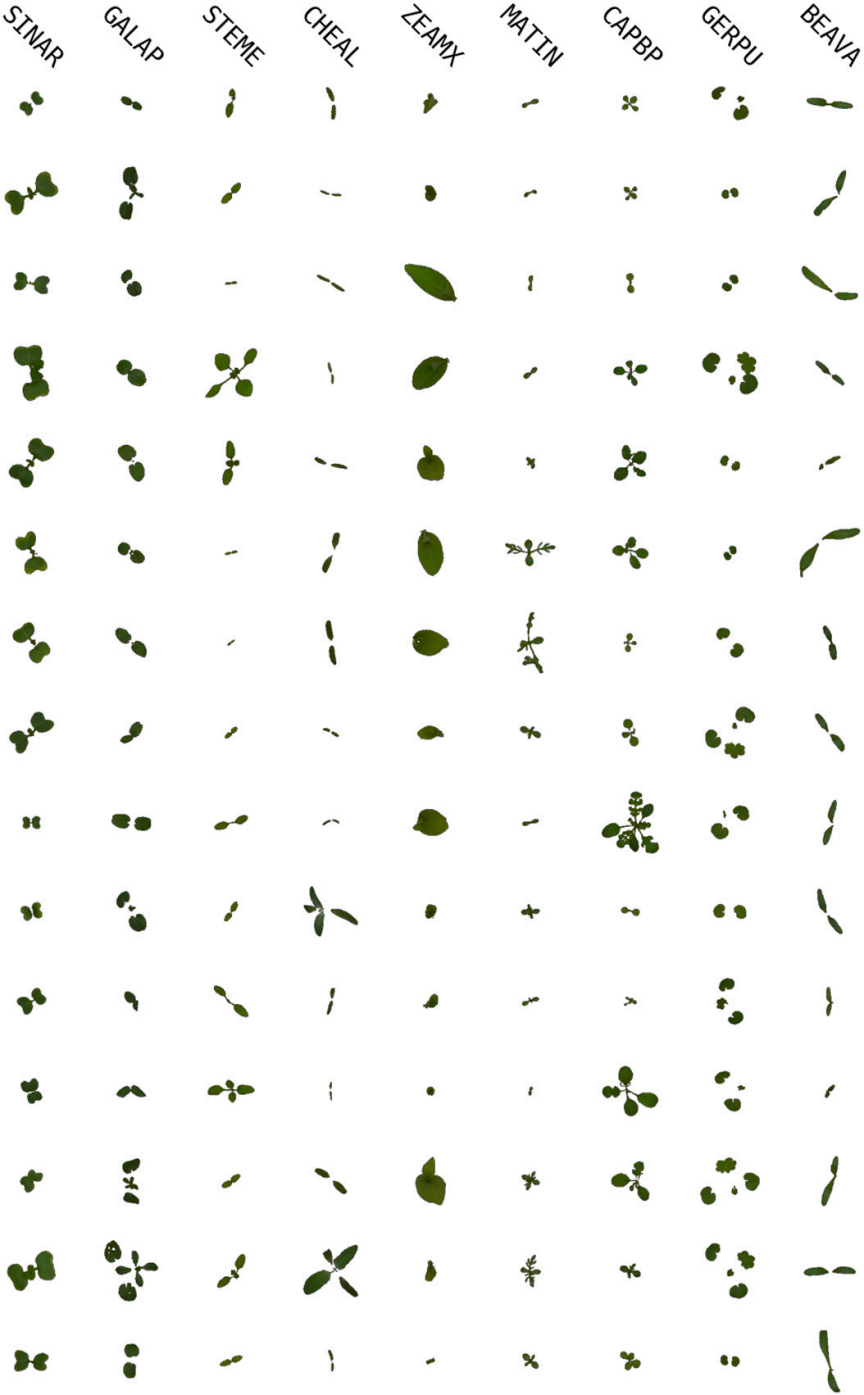
Real data samples from the sPSD. Each column represents a different plant species. With the backgrounds modified from black to white to enhance image contrast. It is worth noting that plant species are consistently identified using the European and Mediterranean Plant Protection Organization (EPPO) coding scheme, as outlined in EPPO 2018, throughout the entire paper.

**Fig. 2.**
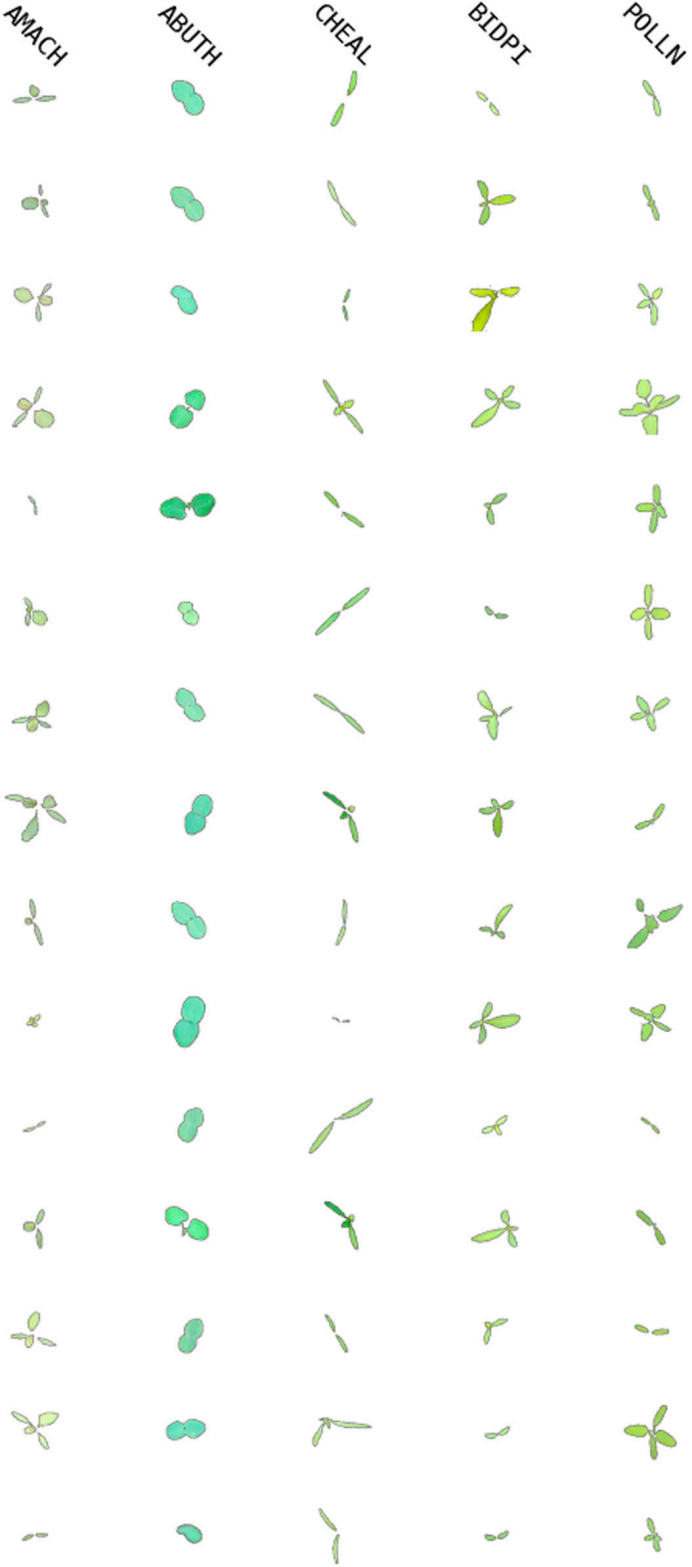
Real data samples from the ISAS dataset. Each column represents a different species, with the backgrounds modified from black to white to enhance the image contrast. Plant species are also consistently identified according to the EPPO coding scheme.

Tables 1 and 2 present an overview of the respective datasets, including species details and sample counts, where we note that plant species are identified using the EPPO coding scheme. Figures 1 and 2 showcase samples from each dataset, with enhanced contrast for better visualization.

**Table 2.** Overview of the plant species and sample count of the ISAS dataset

Weed code	Latin name	Japanese name	No. of samples
AMACH	*Amaranthus hybridus*	Hosoaogeitou	375
ABUTH	*Abutilon theophrasti*	Ichibi	340
BIDPI	*Bidens pilosa var. pilosa*	Kosendangusa	863
POLLN	*Persicaria lapathifolia*	Oinutade	922
CHEAL	*Chenopodium album*	Shiroza	497

### Channel attention mechanism-driven GAN

#### Channel attention block

The SA-GAN [20] adds a self-attention module [21] to improve the ability of both the generator and discriminator to model the global structure, which allows the models to focus on important regions of the image and consider global dependencies, resulting in more realistic and high-quality generated images. However, the self-attention module consumes a large quantity of computational resources and memory. For SA-GAN, the self-attention module is applied to feature maps, which are two-dimensional tensors with spatial dimensions, resulting in a computational complexity of O(*n*^2^*dk*), where *k* is the number of feature maps. However, this method can be computationally expensive, particularly for larger images. In addition, the self-attention module requires a significant amount of memory to store attention scores, which can be prohibitive for certain hardware configurations.

Inspired by SA-GAN, we introduced channel attention module, specifically the squeeze-and-excitation (SE) networks [22], into our GAN architecture. This modification effectively addresses the challenge of weed image generation, where images often exhibit considerable variability due to factors such as lighting conditions, occlusions, and growth stages. Channel attention allows our model to adaptively recalibrate channel-wise feature responses by emphasizing informative features and suppressing less useful ones. This adaptability is crucial in capturing and recreating the nuanced differences between various weed species and their growth stages. Furthermore, the channel attention mechanism is particularly beneficial in dealing with imbalanced datasets common in agricultural contexts, where certain weed species are underrepresented. By employing channel attention, our GAN can more effectively emphasize features distinctive to less common weeds, enhancing the robustness and diversity of the generated images.

**Fig. 3.**
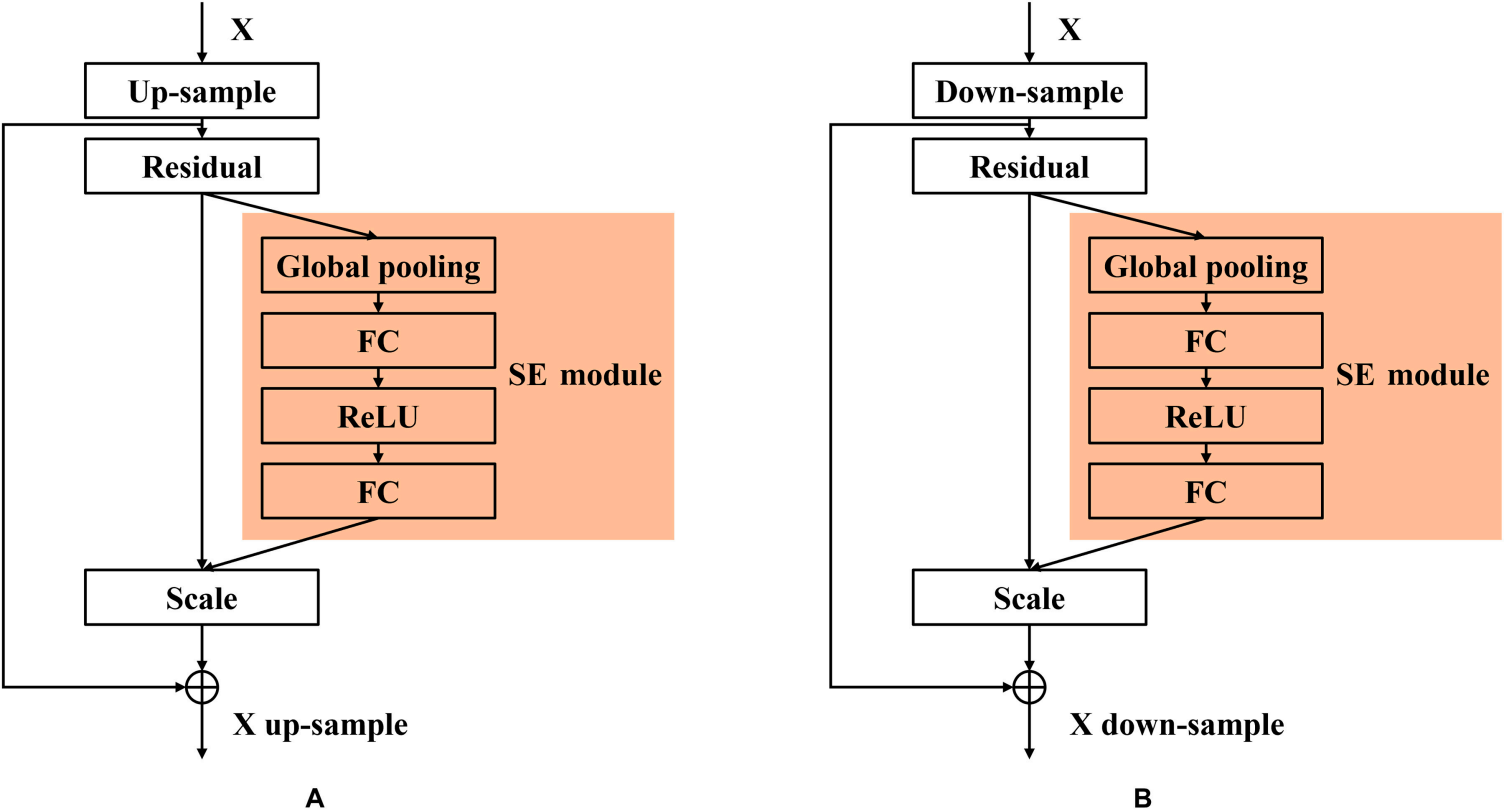
Channel attention module architecture with integrated SE modules. (A) ResNet up-sample block featuring a residual block followed by a SE module. (B) ResNet down-sample block with a SE module integrated after the residual block.

Moreover, while channel attention adds a slightly increase in computational effort, it is more efficient than the self-attention module used in SA-GAN. A SE module (Fig. 3) was introduced in the residual parts of both the generator and discriminator blocks to learn the importance of each feature channel. The SE module-equipped generator and discriminator block architectures, as shown in Fig. 3, filter out less useful feature channels and strengthen the weight of useful channels, thereby yielding better results with manageable computational demands.

**Fig. 4.**
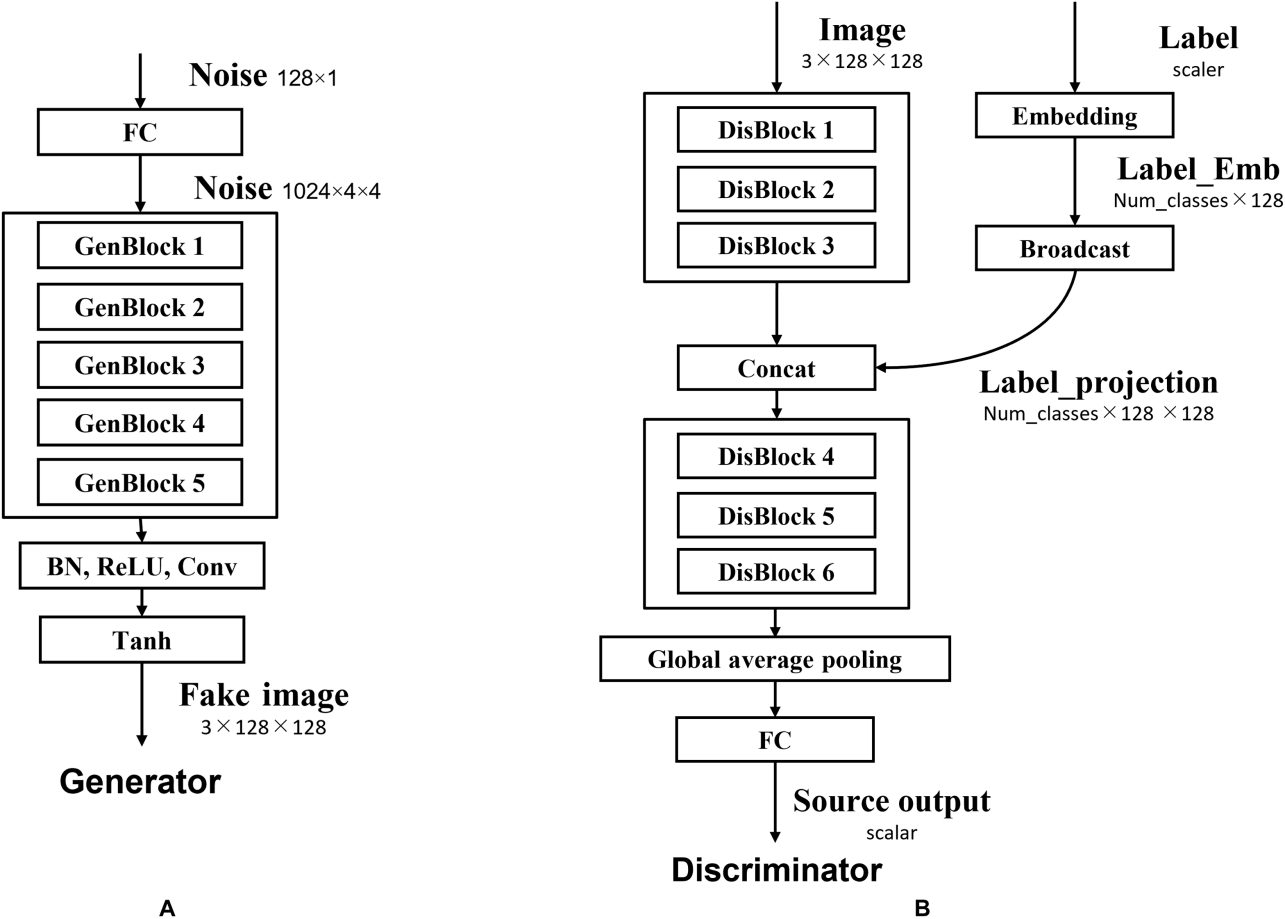
CA-GAN model architecture for the generator and discriminator. (A) Generator—128 × 1 noise input to FC layer, resized to 1,024 × 4 × 4, passed through five generator blocks, followed by BN, ReLU, Conv, and Tanh to output the synthetic image. (B) Discriminator—3 × 128 × 128 image input through three discriminator blocks, label projection (embedding, broadcasting, concatenating) with block output, followed by three more discriminator blocks, global average pooling, and FC layer to obtain the source output.

#### Architecture description

As a baseline, we employed the SN-GAN architecture of Miyato et al*.* [23], which uses a hinge loss [24] GAN objective. We provided class information to the generator with class-conditional BatchNorm [25] and to the discriminator with projection [11].

In addition to class-conditional BatchNorm, we further adopted a latent mapping network as shown in Fig. 4, similar to Karras et al*.* [26], to disentangle the latent space and visual features by producing the latent input vector within the intermediate vector. In practice, we first embedded the class information into codes, concatenated these codes with noise vectors, and sent them to a five-layer mapping network.

Our model adopts several architectural choices from Brock et al*.* [27] including our nomenclature for describing the network width. Network width is determined by the product of a channel multiplier (*ch*) and layer-wise constant. For the generator, layer-wise constants were set as [16, 8, 4, 2, 1] for a 128 × 128 resolution input. The width of the *i*^th^ layer is calculated as the product of *ch* and the *i*^th^ constant, and all layers prior to the residual network in the generator use the multiplier of the initial layer, which we denote as *ch*_0_. In our model, *ch* is set to 64. Similarly, for the discriminator, the corresponding *ch* are [1, 2, 4, 8, 16, 16].

### Evaluation metrics

Visual inspection and statistical evaluation were performed to evaluate the fidelity of the synthetic data. This approach ensures a holistic understanding of the model’s performance, taking into account both qualitative and quantitative aspects.

#### Class discriminability test

As part of our statistical evaluation, the class discriminability test plays a crucial role. It measures the extent to which synthetic samples accurately represent the intended species. This is achieved by employing a well-trained auxiliary classifier, specifically a ResNet-56 model, which we first trained on the real dataset. Subsequently, we applied this classifier to the synthetic dataset and calculated the discrimination accuracy for each class. The discrimination accuracy of this auxiliary classifier thus reflects how precisely the synthetic sample is classified as a specific species. It is important to note that while the class discriminability test offers insight into the sample recognition capability of a particular classifier, it also indirectly serves as a reference for the GAN model’s ability to produce distinct species.

#### Fréchet inception distance

To quantify the similarity between synthetic and real datasets, we utilize the FID score. FID has demonstrated a strong correlation with human assessments of synthetic sample quality. It is calculated by measuring the Fréchet distance between two Gaussian distributions, which are fitted to the feature representations extracted from an inception network.

#### ResNet score

Complementing the above metrics, we introduce the ResNet score (RS) as an innovative metric, particularly focused on evaluating the diversity and clarity of the generated images. Inspired by the inception score (IS), RS adapts its methodology to use a ResNet architecture. Considering our dataset’s specific focus on various plant species, RS leverages the ability of our ResNet model (trained on the same dataset) to provide categorical probability distributions for each generated image. RS assesses two key aspects: the clarity of classification into distinct categories (indicative of the model’s confidence) and the diversity of images across these categories. It calculates the entropy of predicted class distributions for each image (for clarity) and the average entropy across all images (for diversity), ultimately providing a comprehensive metric for our model’s performance evaluation.

## Results

We trained two separate models: one for the benchmark sPSD and one for the auxiliary ISAS dataset. Each model was trained with a batch size of 50 for 70,000 iterations (i.e., with the generator updated 70,000 times in total) using a single GeForce RTX 3090 GPU. We used the Adam optimizer with *β*_1_ = 0.5, *β*_2_ = 0.9, and a learning rate of 2 × 10^−4^. We applied the hinge loss instead of the Wasserstein loss. Besides these two, we also trained a model for the auxiliary ISAS dataset while with background not removed from the original image data to showcase the influence of the background to the model performance.

We also reproduced the state-of-the-art WacGAN-info [16] and SN-GAN [23] networks for comparison. For SN-GAN, we applied the same settings to obtain fair cross-sectional comparison results: When training WacGAN-info [16], the training settings were the same as those given in the original paper [16], i.e., crit repeat was set to 5, the generator learning rate was set to *lr* = 0.001, the discriminator learning rate was set to 0.0002, and the info term coefficient was set to 2. The classification loss function term coefficient *w_C_* = 7.5, info loss term coefficient *w_I_* = 15, gradient penalty coefficient *λ* = 10, and *Leaky ReLU slope* =  −0.2 were applied.

**Fig. 5.**
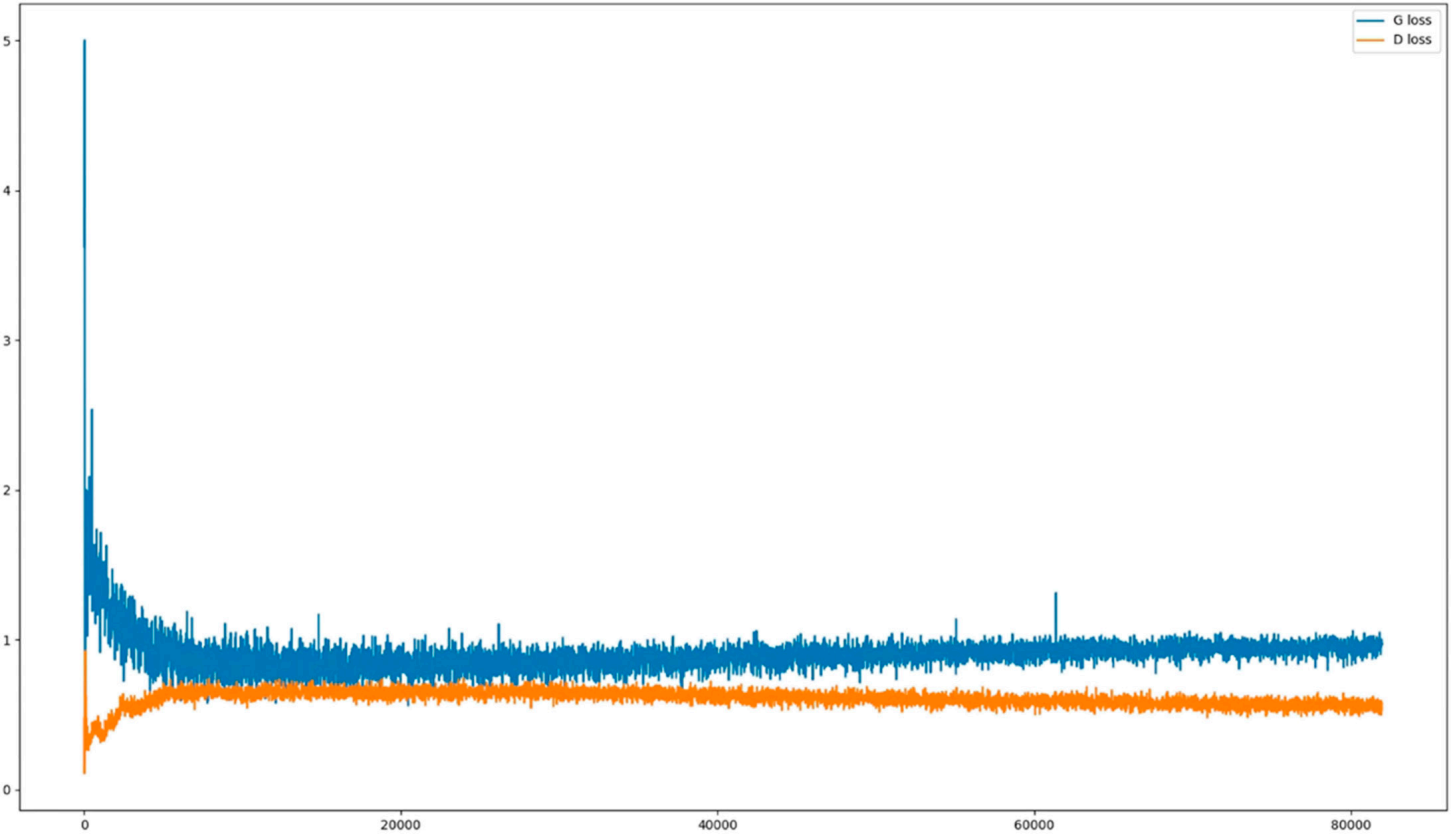
Loss curve of CA-GAN model training process on the sPSD. The blue line represents the generator loss, while the orange line represents the discriminator loss.

Based on the approach of Sun et al*.* [28], we similarly present our results, showcasing the training loss curve of CA-GAN on the sPSD here in Fig. 5. We observed that both the generator and discriminator losses converge rapidly within the first 5,000 iterations and gradually stabilize after 10,000 iterations. It is noteworthy that the convergence rate and time of the loss curves primarily depend on the equilibrium between the generator and the discriminator. Consequently, we set the training ratio of the discriminator to generator at 2:1 and initialized the model weights using the Xavier method [29].

Upon the completion of training, the CA-GAN model was employed to generate 1,000 synthetic samples per class, which were subsequently utilized as an additional test set for evaluating class discriminability and FID score.

### Visual inspection of synthetic data

**Fig. 6.**
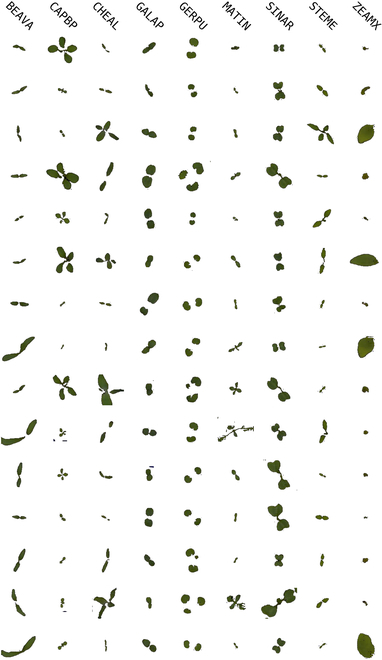
Examples of CA-GAN synthetic data trained on the sPSD. Each row represents a fixed noise vector input, and different columns represent different weed species.

**Fig. 7.**
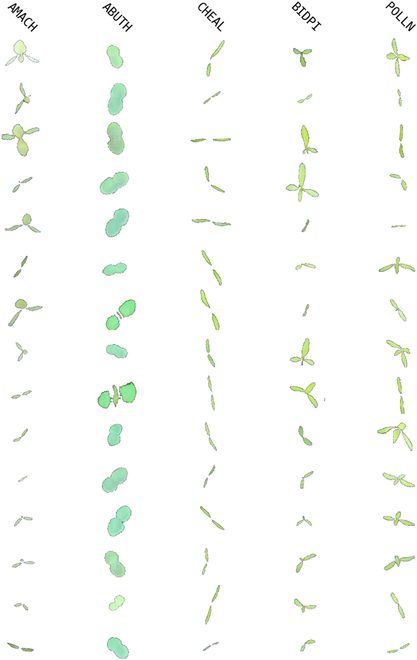
Examples of CA-GAN synthetic data trained on the ISAS dataset. Each row represents a fixed noise vector input, and different columns represent different weed species.

Figures 6 and 7 show examples of synthetic data randomly selected from the synthetic dataset. By visually inspecting the synthetic data samples, it is evident that even when using the same noise input, the proposed GAN model could produce visually different samples among different types of weeds.

**Fig. 8.**
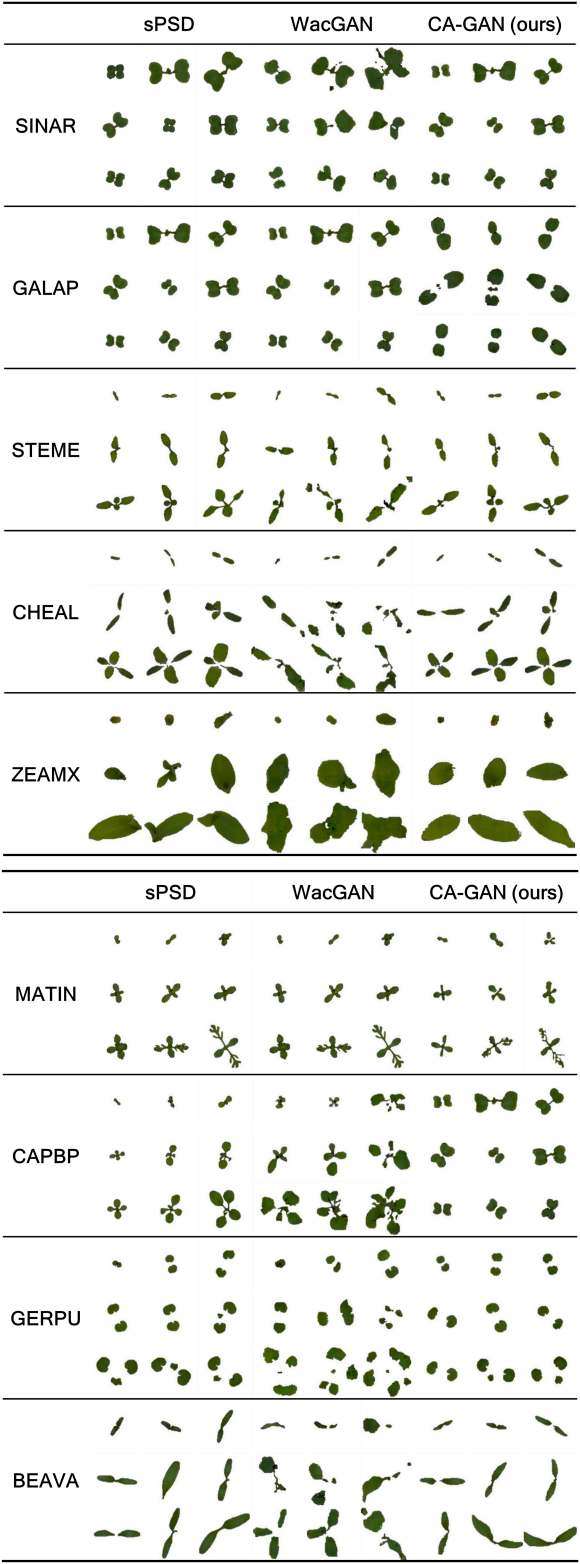
Examples of plant seedlings from sPSD, WacGAN, and CA-GAN, arranged from top to bottom with each species’ frame. Nine samples for each species were generated using identical fixed random noise vectors, with only the class encoding being altered. To enhance contrast, the background of all samples has been switched from black to white.

In Fig. 8, synthetic examples of plant seedlings generated by the CA-GAN model are shown. The figure also provides real examples of plant seedlings and synthetic samples generated by the WacGAN-info model as references for a visual evaluation. Nine samples were generated for each species, all using the same fixed random noise vectors with class-encoding changes. This demonstrated the CA-GAN model’s ability to generate visually distinct samples for each of the different species present in the sPSD, as the appearance of the samples varies across each column.

The synthetic samples consisted of multiple leaves arranged around the center of the plant. In comparison to the samples generated by [16], the CA-GAN model shows the capability to generate more intricate shape and textural features. For instance, our CA-GAN model could clearly generate the edges of the leaves, achieving a realistic effect, whereas WacGAN-info generated samples that often cannot perfectly mimic the details of real data, especially in terms of generating independent leaves, resulting in blurry edges or even merging them together. Meanwhile, WacGAN-info sometimes generates incorrect numbers of leaves (e.g., GERPU), which to some extent indicates that it cannot accurately generate the plants of a stipulated species, whereas CA-GAN does not present this fault. Furthermore, plants generated by WacGAN-info contain a large number of pixel errors, whereas our CA-GAN hardly has any such errors and could realistically simulate the texture of leaves.

### Visualization of channel attention mechanism

**Fig. 9.**
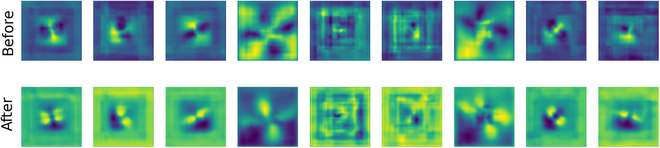
Visualization of feature maps before and after the channel attention mechanism (GenBlock 5 as an example). The first row represents the feature maps before the channel attention mechanism, while the second row represents the feature maps after the channel attention mechanism. Different columns represent different feature map channels.

We visualized the feature maps both before and after the channel attention mechanism in Fig. 9 to help us in comparing the activations directly and observing the influence of the channel attention mechanism to the generator.

It can be observed quite distinctly that, following the application of channel attention, the model focuses more on generating the plants themselves rather than their surroundings or background, which means that the application of channel attention mechanisms has led to a noticeable shift in the model’s focus, directing more attention toward accurately rendering the plants themselves, while less emphasis is placed on the surrounding environment or background. This highlights the model’s enhanced ability to prioritize and capture the essential features of the subject matter.

### Class discriminability test

**Fig. 10.**
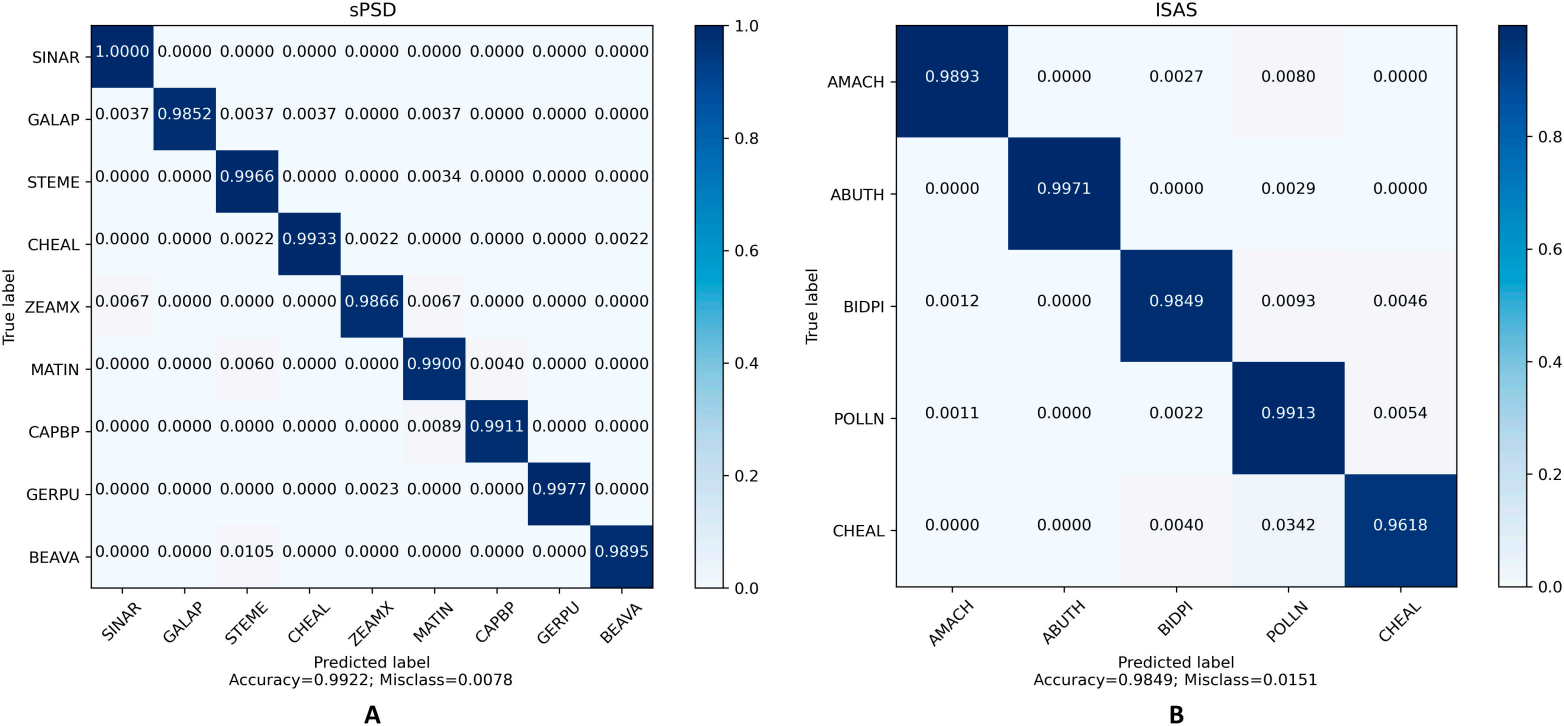
Average confusion matrix, indicating classification accuracy, when evaluating the real samples from the standard sPSD (A) and auxiliary ISAS (B) datasets using the auxiliary classifier, which was trained exclusively on real samples. Each row has been normalized concerning the number of true labels for that specific class.

We also trained an auxiliary classifier on a real dataset to observe the accuracy of the generated data classes when the trained GAN responded to the different class labels. The auxiliary classifier, also based on the ResNet model, achieved 99.22% and 98.49% classification accuracy on the real data of the sPSD and ISAS datasets, respectively (Fig. 10), indicating that the auxiliary classifier obtained from the training was sufficiently accurate to provide classification labels that were close to the ground truth.

**Fig. 11.**
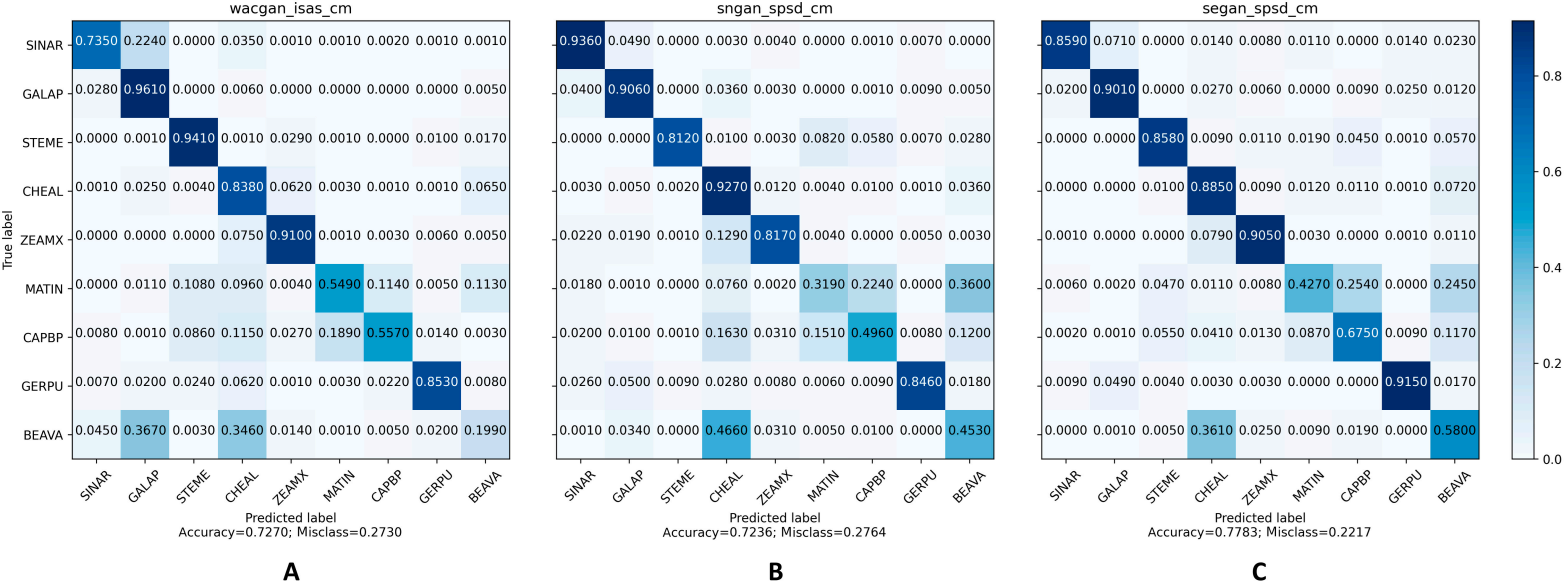
Classification accuracy on the synthetic dataset generated by different GAN models (A: WacGAN-info, B: SN-GAN, C: CA-GAN, our model) using the auxiliary trained classifier on the sPSD.

**Fig. 12.**
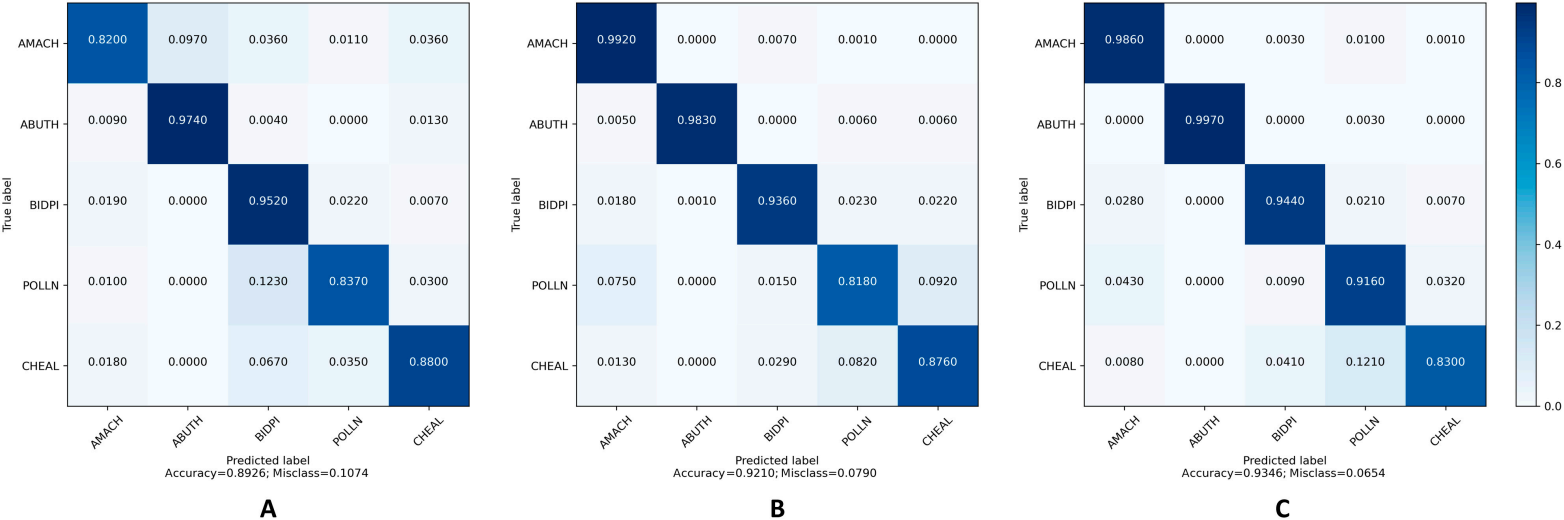
Classification accuracy on the synthetic dataset generated by different GAN models (A: WacGAN-info, B: SN-GAN, C: CA-GAN, our model) using the auxiliary trained classifier on the ISAS dataset.

We then applied the classifier to the synthetic dataset, and Fig. 8 shows the classification results. As shown in Figs. 11 and 12, our CA-GAN network obtained 82.63% and 93.46% classification accuracies on the sPSD and ISAS datasets, respectively, both of which are higher than those of the current state-of-the-art methods.

We additionally trained a classifier on the auxiliary ISAS dataset, this time without removing the background from the image data, to demonstrate the impact of background removal. In this setup, our CA-GAN network achieved a classification accuracy of only 84.00%, significantly lower compared to its performance on the ISAS dataset with background removed. This outcome also supports our hypothesis that including background in the generative process substantially increases model complexity. The model must then account for varying soil textures, moisture levels, and lighting conditions, which can differ greatly across different environments.

### FID score test

The final CA-GAN achieved a minimum FID score of 20.95 on the benchmark sPSD and 24.31 on the auxiliary ISAS dataset. The results showed that CA-GAN performed best on the ISAS dataset while achieving comparable results with the SN-GAN on the sPSD (Table 3).

**Table 3.** Results and comparison with other models

	sPSD			ISAS dataset		
Model	FID score↓	Classification accuracy↑	ResNet score↑	FID score↓	Classification accuracy↑	ResNet score↑
Baseline	-	99.22%	2.30	-	98.49%	4.56
WacGAN-info	53.63	72.70%	8.68	53.31	89.26%	4.94
SN-GAN	19.20	81.77%	8.64	39.77	92.10%	4.94
CA-GAN (proposed)	20.95	82.63%	8.64	24.31	93.46%	4.94

### RS test

The evaluation of our CA-GAN model using the RS has yielded promising results. On the sPSD, our CA-GAN achieved an RS of 8.64, which is on par with the SN-GAN and significantly surpasses the baseline score of 2.30. Although marginally lower than the WACGAN-info’s score of 8.68, the competitive performance of our model demonstrates its efficacy in generating high-quality and diverse images. On the ISAS dataset, our CA-GAN’s RS of 4.97 not only equals the performance of SN-GAN but also slightly exceeds the WACGAN-info’s score of 4.94 while substantially outperforming the baseline score of 4.56 (Table 3).

## Discussion

In this study, we presented the results of experiments testing the use of GANs in weed synthesis. In particular, we explored the use of GANs to generate images of weed species.

One critical aspect of this study was the use of latent space to achieve continuous control during the early growth stages of weeds. Through our experiments, we demonstrated that the generator network could achieve continuity control in the early growth stages of nine representative types of agricultural weeds, excluding grass species, within the latent space. This was performed by selecting two fixed noise vectors, *z*_1_ and *z*_2_, and linearly interpolating them. The result was a continuously varying noise input, which was then fed into the generator. Thus, we obtained generated images that changed smoothly as the input noise gradually changed, demonstrating the continuity of the trained generator network.

**Fig. 13.**
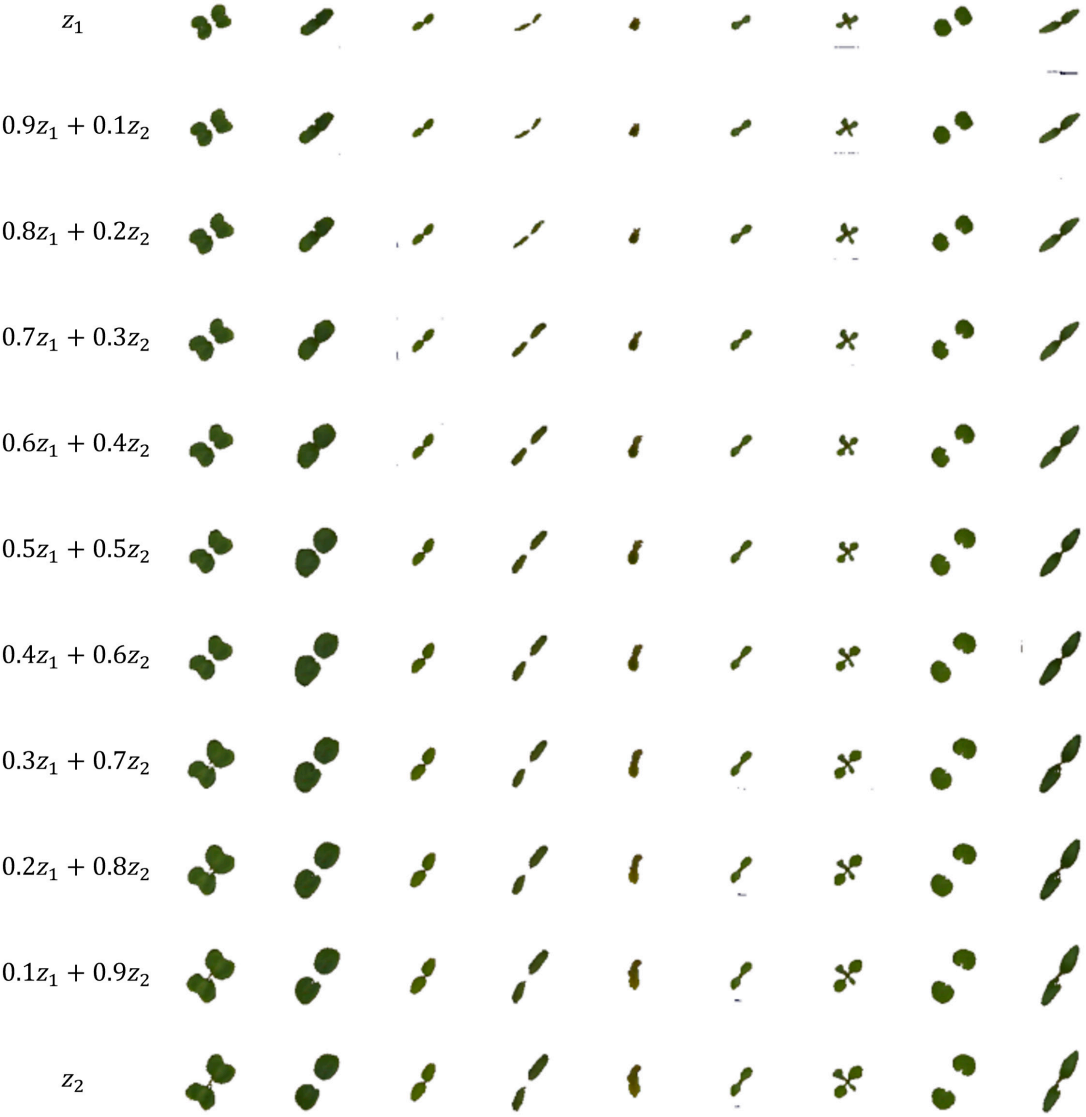
CA-GAN latent space visualization of the sPSD. Different columns represent different species of weeds, while different rows, from top to bottom, represent the continuous variation of the input noise from *z*_1_ to *z*_2_.

Figure 13 shows the results obtained by selecting two fixed noise vectors, *z*_1_ and *z*_2_, linearly interpolating between *z*_1_ and *z*_2_, and inputting the obtained continuously varying noise into the generator. Different columns represent different species of weeds, whereas different rows, from top to bottom, represent continuous variation in the input noise from *z*_1_ to *z*_2_.

This indicates that the continuity feature can be appropriately used to achieve a certain degree of control over the continuity of weed growth. For instance, we can use this feature to generate images of weed species that vary gradually in specific features, such as color, shape, or size. Such features are essential for weed management, as it is crucial to accurately identify weed species and their characteristics.

However, this method of controlling the weed growth stages has significant limitations. For example, we must first determine the noise that represents the earliest and most mature stages of weed growth, which is difficult in practical operations. Therefore, we need to develop a more stable method for controlling the plant growth stages.

In addition, our pipeline achieved good generation results on small datasets through data cleansing and the incorporation of the channel attention module in the GAN network. However, there is still room for improvement in the generation performance of small training samples, such as the ZEAMX variety in the sPSD, which contains fewer than 200 training images. In some areas of the generated images, rough edges and even pixel errors remained. Therefore, more data are required to obtain better generation results, in addition to improving the network.

Our future research should take steps to (a) achieve control over the growth stages and (b) build a fully automated data collection system to obtain a large quantity of high-resolution image data.

## Data Availability

The source code and dataset used in this study can be accessed at https://github.com/UTokyo-FieldPhenomics-Lab/WeedGeneration.
